# Mold Alkaloid Cytochalasin D Modifies the Morphology and Secretion of fMLP-, LPS-, or PMA-Stimulated Neutrophils upon Adhesion to Fibronectin

**DOI:** 10.1155/2017/4308684

**Published:** 2017-06-27

**Authors:** Svetlana I. Galkina, Natalia V. Fedorova, Marina V. Serebryakova, Evgenii A. Arifulin, Vladimir I. Stadnichuk, Ludmila A. Baratova, Galina F. Sud'ina

**Affiliations:** ^1^A. N. Belozersky Institute of Physico-Chemical Biology, Lomonosov Moscow State University, Leninskie Gory, Moscow 119234, Russia; ^**2**^ Physical Department, Lomonosov Moscow State University, Leninskie Gory, Moscow 119234, Russia

## Abstract

Neutrophils play an essential role in innate immunity due to their ability to migrate into infected tissues and kill microbes with bactericides located in their secretory granules. Neutrophil transmigration and degranulation are tightly regulated by actin cytoskeleton. Invading pathogens produce alkaloids that cause the depolymerization of actin, such as the mold alkaloid cytochalasin D. We studied the effect of cytochalasin D on the morphology and secretion of fMLP-, LPS-, or PMA-stimulated human neutrophils upon adhesion to fibronectin. Electron microscopy showed that the morphology of the neutrophils adherent to fibronectin in the presence of various stimuli differed. But in the presence of cytochalasin D, all stimulated neutrophils exhibited a uniform nonspread shape and developed thread-like membrane tubulovesicular extensions (cytonemes) measuring 200 nm in diameter. Simultaneous detection of neutrophil secretory products by mass spectrometry showed that all tested stimuli caused the secretion of MMP-9, a key enzyme in the neutrophil migration. Cytochalasin D impaired the MMP-9 secretion but initiated the release of cathepsin G and other granular bactericides, proinflammatory agents. The release of bactericides apparently occurs through the formation, shedding, and lysis of cytonemes. The production of alkaloids which modify neutrophil responses to stimulation via actin depolymerization may be part of the strategy of pathogen invasion.

## 1. Introduction

Neutrophils, a critical component of innate immunity, play an important role in host defense against bacterial pathogens due to their capacity to migrate into infected tissues and phagocytose and kill bacteria. Neutrophils destroy bacteria by releasing bactericidal agents from intracellular secretory granules into the phagosome or to the cell exterior [1, 2]. The release of granular bactericides from the cell happens also upon neutrophil adhesion to endothelium, which is the first step of neutrophil migration to infected tissues [3, 4]. Transendothelial migration of neutrophils strongly depends on cytoskeletal remodeling and contractility [5]. Degranulation or exocytosis, cellular processes based on membrane dynamics, is also tightly associated with actin cytoskeleton [6]. Invading bacterial or fungal pathogens produce alkaloids, such as mold alkaloid cytochalasin D, *Streptomyces staurosporeus* alkaloid staurosporine, or *Clostridium difficile* binary toxin (CDT), which cause depolymerization of the actin cytoskeleton in host cells via different mechanisms [7–9]. These alkaloids can significantly affect cellular responses of stimulated neutrophils.

Neutrophils contain primary (azurofil), secondary (specific), and tertiary (gelatinase) granules, as well as secretory vesicles [1]. The composition of granules of different types largely overlaps [2]. Nevertheless, cathepsin G and other antibacterial agents are contained mainly in the primary granules. Lactoferrin and lipocalin are considered components of the secondary granules. Metalloproteinases concentrated in tertiary granules of neutrophils. Different signaling pathways trigger degranulation process in different types of granules. To induce degranulation of primary granules, secretory stimuli such as *N*-formylmethionyl-leucyl-phenylalanine (fMLP) or phorbol 12-miristate 13-acetate (PMA), an activator of protein kinase C, are usually applied in combination with cytochalasin D or sponge alkaloid latrunculin A, another actin-depolymerizing agent [10, 11]. The mechanism of action of microbial alkaloids on neutrophil secretory processes remains unknown. It is supposed that the destruction of the cytoskeleton facilitates the primary granule access to the plasma membrane enabling fusion and release of their content. However, recent data indicate that neutrophil secretion occurs through various mechanisms, including budding of secretory membrane vesicles from the plasma membrane [12–16] and cumulative or compound exocytosis, when the granules fuse with each other intracellularly and share a common opening in the plasma membrane to release their contents [17].

The effect of actin-depolymerizing alkaloids on neutrophil adhesion and accompanying secretion is much less studied. Previous scanning electron microscopy studies revealed that the adhesion of resting neutrophils to fibronectin in the presence of cytochalasin D, latrunculin A, or staurosporine was accompanied by the formation of cytonemes, thread-like membrane tubulovesicular or tubular extensions measuring 200 nm in diameter [18–21]. Cytonemes, which may be longer than the diameters of several cells, are able to establish direct contact of neutrophils with the cells or microbes at a distance [19, 22]. Clostridium difficile toxin (CDT), which induces the depolymerization of the actin cytoskeleton via ADP-ribosylation [7], causes the formation of cytoneme-like protrusions at the surface of intestinal epithelial cells which wrap and embed bacteria *Clostridia* [23, 24].

We observed the formation of cytonemes in human neutrophils upon the inhibition of vacuolar-type ATPase, the blocking of glucose metabolism, the inhibition of GTPase dynamin, or in the presence of nitric oxide donor diethylamine NONOate or cytochalasin D [18, 21, 22, 25–27]. Proteome analysis has revealed that (i) the cytonemes contain bactericides of primary and secondary secretory granules, cytoplasmic proteins such as actin cytoskeleton and S100 proteins, and energy-metabolizing (presumably glycolytic) enzymes and (ii) the content of cytonemes does not depend on agent inducing their formation [20, 21]. However, it is not known whether cytoneme formation occurs and plays a role in the process of neutrophil activation and degranulation. In this work, we studied the effect of cytochalasin D on the morphology and secretion of fMLP-, LPS- (lipopolysaccharide from *Salmonella enterica* serovar Typhimurium-), or PMA-stimulated neutrophils upon adhesion to fibronectin. We applied scanning and transmission electron microscopy (SEM and TEM, resp.) to study the external and internal morphology of neutrophils. In parallel, we examined the composition of neutrophil secretory products using mass spectrometry to identify proteins in the extracellular medium.

## 2. Materials and Methods

### 2.1. Materials

Ficoll-Paque was purchased from Pharmacia (Uppsala, Sweden). Fibronectin was from Calbiochem (La Jolla, USA). Bicarbonate-free Hank's solution, Ca^+^-free Dulbecco PBS, fMLP, PMA, LPS (lipopolysaccharide from *Salmonella enterica* serovar Typhimurium), E64, and cytochalasin D were obtained from Sigma (Steinheim, Germany); trypsin from Promega; PMSF from Biomedical (Illkirch, France); and Coomassie Brilliant Blue G-250 from Serva.

### 2.2. Neutrophil Isolation

Neutrophils were isolated from citrate-anticoagulated freshly drawn donor blood as previously described [20, 21]. Human venous blood was collected from healthy volunteers according to the recommendations of the Institutional Ethics Committee of the A. N. Belozersky Institute of Physico-Chemical Biology. Erythrocytes were removed by dextran sedimentation (6% final concentration). Neutrophils were isolated by density centrifugation on Ficoll-Paque (1.077) followed by a hypotonic lysis of the remaining red blood cells. After washing, the neutrophils were suspended and stored prior to the experiment in the Ca^+^-free Dulbecco PBS.

### 2.3. Adhesion of Neutrophils to Fibronectin-Coated Coverslips

To study the morphology of the cells with electron microscopy, neutrophils were plated to the fibronectin-coated coverslips. The clean coverslips were incubated in a solution of fibronectin (5 *μ*g/mL) for 2 hours at room temperature, washed, and placed in a 40 mm Petri dish. Neutrophils (3 × 10^6^ cells/2 mL) attached to fibronectin-coated coverslips in the bicarbonate-free Hank's solution containing 10 mM HEPES (pH 7.35) during 20 minutes at 37°C. Cytochalasin D (10 *μ*g/mL), fMLP (1 *μ*M), PMA (0.1 *μ*M), or LPS (20 *μ*g/mL) was added to the neutrophils immediately before the experiment. At the end of the incubation time, the glass with the attached neutrophils was transferred to the fixation solution. The cells were then prepared for examination by electron microscopy.

### 2.4. Determination of the Protein Composition of Neutrophil Secretion

In order to study the protein composition of neutrophil secretion accompanying cell adhesion, we placed the neutrophils in plastic 6-well plates coated with fibronectin. Beforehand, 2 mL of fibronectin solution (5 *μ*g/mL) was added to each well. After two hours of incubation at room temperature, the wells were washed with buffer. Neutrophils (3 × 10^6^ cells/2 mL) were plated to the wells in the bicarbonate-free Hank's HEPES solution during 20 minutes at 37°C. The peptide fMLP, PMA, LPS, or cytochalasin D was added to the cells immediately before plating. After the incubation, the extracellular medium from all the wells was sampled. The inhibitors of metalloproteinase, serine and cysteine proteinase, and myeloperoxidase (EDTA, 5 mM; PMSF, 200 *μ*M; E64, 10 *μ*M; sodium azide, 0.025%) were added to the samples to prevent protein proteolysis during further processing—centrifugation, extraction, and so forth. Nonattached neutrophils were removed by centrifugation. Samples from six analogous wells were combined.

### 2.5. Concentration and Separation of Proteins by Polyacrylamide Gel Electrophoresis

Proteins were extracted from the samples of extracellular medium with a chloroform-methanol mixture (2 : 1). An equal volume of chloroform-methanol mixture was added to the sample, and the resulting mixture was shaken at 4°C for 30 minutes. To separate the phases, the mixture was centrifuged for 20 min at 11000*g*. In previous works, we found out that practically all the proteins fall into the chloroform fraction as a result of this procedure [20, 21]. The chloroform phase was sampled, and after the evaporation of the solvent, the proteins were subjected to electrophoresis.

To separate the proteins, a one-dimensional polyacrylamide gel electrophoresis in the presence of sodium dodecyl sulphate (SDS-PAGE) was used [28]. Electrophoresis was performed in a 15% gel using Mini-PROTEAN 3 Cell (Bio-Rad). Prior electrophoresis aliquots of the samples were boiled for 3 minutes in lysis buffer (30 mM Tris-HCl (pH 6.8), 1% SDS, 3 M urea, 10% glycerol, and 0.02% bromophenol blue). The gels were stained with 0.22% Coomassie Brilliant Blue G-250.

### 2.6. Identification of Proteins by Mass Spectrometry and Preparation of Samples

After carrying out the electrophoresis, a piece of gel with a size of 1 × 1 mm^2^ was cut from each Coomassie-stained protein band and trypsin hydrolysis of the protein was performed directly in the gel. To this end, pieces of gel were washed twice with 100 *μ*L of 40% acetonitrile in 100 mM NH_4_HCO_3_ (pH 7.5) for 30 min at 37°C and dehydrated with 100 *μ*L acetonitrile. The samples were air dried and then incubated with 4 *μ*L of modified trypsin (12 *μ*g/mL, Promega) in 50 mM NH_4_HCO_3_ for 6 h at 37°C. To recover the resulting peptides, the samples were incubated for 30 minutes with 6 *μ*L of a solution of trifluoroacetic acid in 10% acetonitrile. The peptides were then analyzed by mass spectrometry. To this end, 1 *μ*L of the sample in a mixture with 0.3 *μ*L of 2,5-dihydroxybenzoic acid (20 mg/mL in 20% acetonitrile and 0.5% trifluoroacetic acid) was dropped onto the steel target and allowed to air dry.

A MALDI-time of flight (ToF) mass spectrometer (Ultraflex II Bruker, Germany) equipped with a neodymium (Nd) laser was used for laser desorption ionization mass spectrometry with matrix activation (MALDI-MS) and tandem mass spectrometry (MS/MS). The [MH]^+^ molecular ions were measured in reflector mode. The accuracy of mass peak measurement was within 0.005%. Mascot software (www.matrixscience.com) and the mammalian protein database of NCBL were used to identify proteins by searching for a peptide fingerprint with the indicated accuracy. The search allowed for the possible oxidation of methionine by environmental oxygen and the modification of cysteine with acrylamide. Protein matches were considered significant (*p* < 0.05) with a score > 71.

### 2.7. Scanning Electron Microscopy Technique

Scanning electron microscope Camscan S-2 was used to study the morphology of fibronectin-attached neutrophils. The neutrophils attached to the coverslips were fixed in 2.5% glutaraldehyde in the Ca^2+^- and Mg^2+^-free Hank's HEPES buffer (pH 7.3) containing inhibitors of metalloproteinases (5 mM EDTA) and serine proteases (0.5 mM PMSF). The cells were further fixed in a 1% solution of osmium tetroxide in 0.1 M sodium cacodylate and 0.1 M sucrose at pH 7.3. After dehydration in graded acetone (10–100%), the cells were dried in a Balzers apparatus with liquid CO_2_ as a transitional fluid, coated with sputtered gold-palladium and examined at 15 kV.

### 2.8. Transmission Electron Microscopy Technique

Transmission electron microscope JEM-1400 was used to observe the internal morphology of neutrophils. The cells were fixed in the same way as described for scanning electron microscopy. Fixed samples were dehydrated in the usual way (70% ethanol containing 2% uranyl acetate), embedded in Epon 812 (Fluka), cut into ultrathin sections with a Reichert Ultra Сut III, and stained with lead citrate.

### 2.9. Actin Cytoskeleton Staining

Fluorescent and phase-contrast images of cells were made using a Zeiss Axiovert 200M microscope. To study the structure of the actin cytoskeleton, neutrophils on cover glasses were fixed in 4% paraformaldehyde the Ca^2+^- and Mg^2+^-free Hank's HEPES buffer containing 5 mM EDTA (pH 7.3). For permeabilization, the cells were incubated for 10 minutes in a 0.1% Triton X-100 solution. Actin was stained with FITC phalloidin.

## 3. Results

### 3.1. Effect of Cytochalasin D on the Morphology of the Neutrophils Attached to Fibronectin in the Presence of Secretory Stimuli

We studied the morphology and composition of neutrophil secretion during the attachment of cells to a solid substrate coated with fibronectin, an extracellular matrix protein. The adhesion to the substrate is an activating factor for cells, and the degree of activation depends on the substrate. During the attachment of neutrophils to fibronectin, the cells are activated much less than when attached to pure glass or plastic [29, 30]. We compared the morphology of neutrophils attached to fibronectin in the presence of fMLP, PMA, or LPS taken alone or in the presence of secretion stimuli in combination with cytochalasin D.

**Figure 1:**
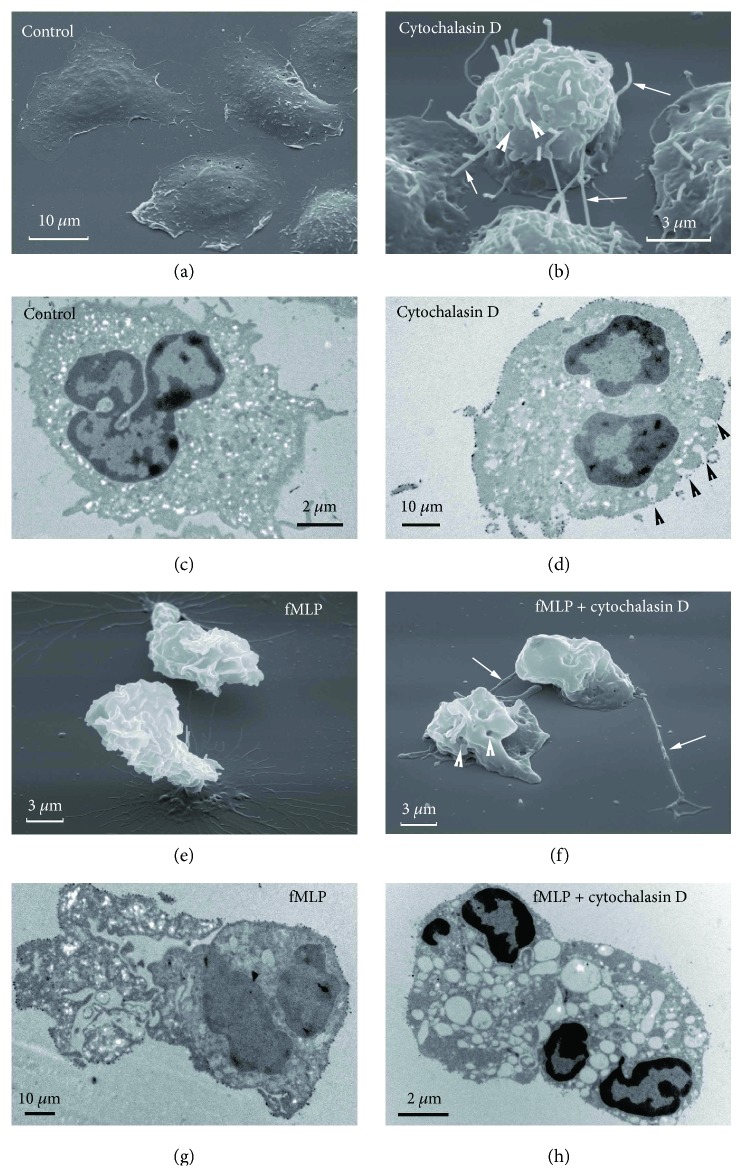
Effect of cytochalasin D on the external and internal morphology of control (resting) and fMLP-treated neutrophils upon adhesion to fibronectin. SEM (a, b, e, f) and TEM (c, d, g, h) images of neutrophils that were attached to fibronectin-coated substrata over a period of 20 min under the control conditions (a, c), in the presence of 10 *μ*M cytochalasin D (b, d) or in the presence of 1 *μ*M fMLP, taken separately (e, g) or together with 10 *μ*M cytochalasin D (f, h). The white arrows point to the cytonemes. Arrowheads (white for SEM and black for TEM images) indicate invaginations of the plasma membrane. Pictures represent typical images observed in two independent experiments.

SEM data demonstrated that the control (resting) neutrophils spread during attachment to the substrate (Figure 1(a)). But, as shown in previous works [18, 20, 21], in the presence of cytochalasin D, resting neutrophils did not spread and numerous filiform tubulovesicular protrusions (cytonemes) appeared on their plasma membrane. The diameter of these protrusions fluctuated within 150–200 nm (Figure 1(b), white arrows). Neutrophils were directly contacted with neighboring cells and substrates through cytonemes. Another hallmark of cells treated with cytochalasin D was the presence of plasma membrane invaginations that were detected with SEM (Figure 1(b), white arrowheads) and TEM data (Figure 1(d), black arrowheads). These invaginations were single depressions with a diameter approximately equal to the diameter of the cytoneme and depth reaching 500 nm [21]. TEM examination of the internal morphology of the cytochalasin D-treated cells showed no significant difference in comparison to control cells with the exception of the previously mentioned invaginations in the plasma membrane (Figures 1(c) and 1(d)).

In contrast to the control cells, neutrophils attached to fibronectin-coated substrata in the presence of the peptide fMLP did not spread and possessed multiple ruffles on their surface (Figure 1(e)). TEM observation of a thin section made through the middle of the cell also demonstrated the many winding folds of the cell surface, indicating increased overall cell surface area (Figure 1(g)). This increase could result from incorporation of secretory vesicles possessing abundant membrane into the plasma membrane of neutrophils. We concluded based on previous data that in vitro stimulation of neutrophils in suspension with fMLP leads to a rapid and almost complete discharge of secretory vesicles without significant release of granules [1, 31].

SEM images of neutrophils that were stimulated with combination of fMLP and cytochalasin D (Figure 1(f)) resembled images of cytochalasin D-treated neutrophils (Figure 1(b)). In this case, we observed nonspread cells with a small number of cytonemes and invaginations that often had a swollen appearance. Phase-contrast observation (data not shown) confirmed that the formation of cytonemes in this case occurred simultaneously with their swelling and shedding from the cells and lysis. Within the cells, the combination of fMLP and cytochalasin D induced the formation of multiple large vacuoles as was revealed by TEM (Figure 1(h)). The formation of vacuoles may be due to the fact that the cytochalasin D stimulated compound or cumulative exocytosis, the fusion of secretory granules with each other inside the cells before the fusion with the plasma membrane [17].

**Figure 2 fig2:**
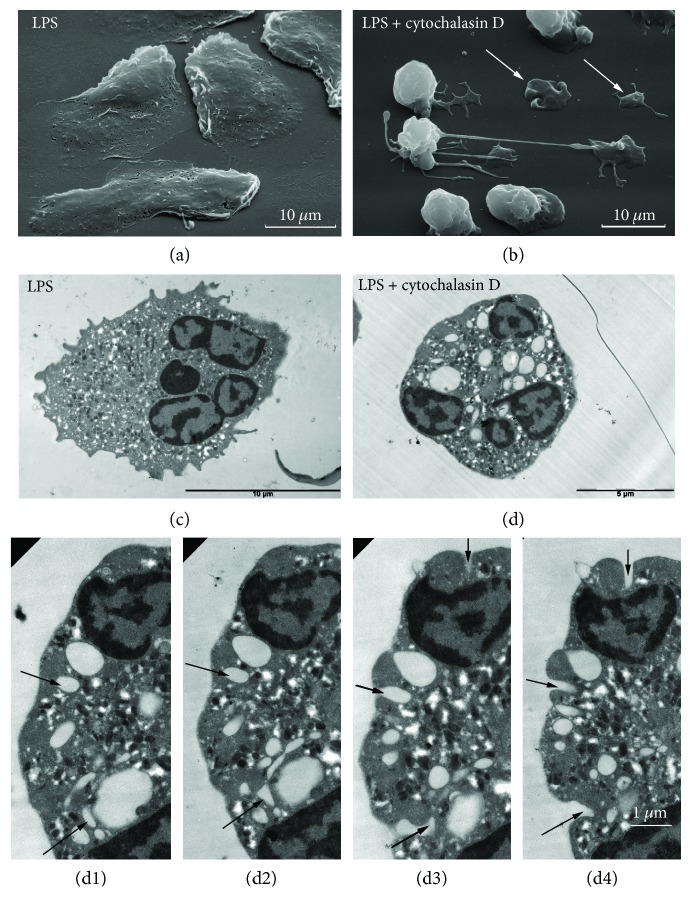
Effect of cytochalasin D on the external and internal morphology of LPS-treated neutrophils upon adhesion to fibronectin. SEM (a, b) and TEM (c, d) images of neutrophils that were attached to fibronectin-coated substrata over a period of 20 min in the presence of 20 *μ*M LPS, taken alone (a, c) or together with 10 *μ*M cytochalasin D (b, d) and TEM images of the thin seral sections of the cell that was attached to substrata in the presence of 20 *μ*M LPS together with 10 *μ*M cytochalasin D (d1–d4). White arrows indicate cytoplasm islands. Black arrows indicate the common exits to the exterior of intracellular vacuoles. Pictures represent typical images observed in two independent experiments.

LPS (lipopolysaccharides of outer membrane of Gram-negative bacteria) or endotoxin is the major inducer of host responses to Gram-negative organisms and effective stimulator of innate immunity, particularly cells of myeloid lineage [32]. The external and internal morphology of cells attached to fibronectin in the presence of LPS resemble the morphology of the control cells (Figures 2(a) and 2(c), resp.). Neutrophils attached to fibronectin in the presence of both LPS and cytochalasin D displayed similar morphology to cytochalasin D-treated cells with plasma membrane invaginations and cytonemes (Figure 2(b)). In these conditions, we observed the formation of cytoplasm islands at the tips of cytonemes. The surfaces of these islands possessed membrane invaginations typical for cytochalasin D-treated cells. We suppose that cytochalasin D in combination with LPS initiated an outward secretory stream that manifested as cytoneme growth. Attachment of cytoneme tips lead to the formation of cytoplasm islands. We do not exclude that migration of activated neutrophils through the gaps between the cells can be carried out through cytonemes via the same mechanism.

TEM data revealed that treatment of neutrophils with LPS in combination with cytochalasin D initiated the formation of multiple intracellular vacuoles (Figure 2(d)). Inspection of thin serial sections through the cells revealed that some of these vacuoles were interconnected and had a common opening to the cell exterior (Figures 2(d1)–2(d4)). The common openings corresponded to cytochalasin D-induced plasma membrane invaginations in size and appearance.

**Figure 3:**
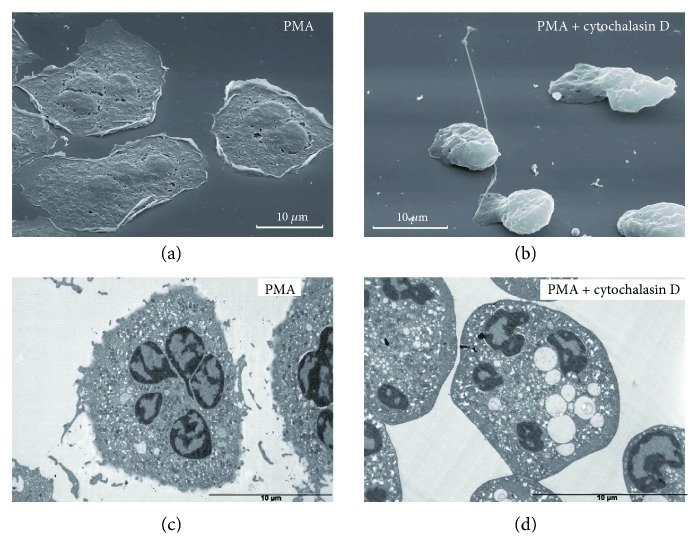
Effect of cytochalasin D on the external and internal morphology of PMA-treated neutrophils upon adhesion to fibronectin. SEM (a, b) and TEM (c, d) images of neutrophils that were attached to fibronectin-coated substrata over a period of 20 min in the presence of 0,1 *μ*M PMA, taken separately (a, c) or together with 10 *μ*M cytochalasin D (b, d). Pictures represent typical images observed in two independent experiments.

It is shown that PMA induces the complete release of gelatinase granules from suspended neutrophils [33]. Neutrophils attached to fibronectin in the presence of PMA were characterized by firm attachment and spreading (Figure 3(a)). The internal morphology of PMA-treated cells resembled that of the control cells (Figure 3(c)). Neutrophils attached to fibronectin in the presence of both PMA and cytochalasin D displayed similar morphology to cytochalasin D-treated cells. Only fragments of cytonemes and some plasma membrane invaginations were observed on the cell surface, indicating that destructive processes occurred in parallel with the formation of these membrane structures (Figure 3(b)). TEM data revealed that the combination of PMA and cytochalasin D induced the formation of large vacuoles inside the neutrophils (Figure 3(d)).

### 3.2. Effect of Cytochalasin D on the Composition of Neutrophil Secretion upon Adhesion to Fibronectin in the Presence of fMLP, LPS, or PMA

The existence of various mechanisms of exocytosis in neutrophils does not allow for developing a common method for measuring this process. When granules fuse with the plasma membrane [10], the intensity of degranulation can be estimated by the appearance of specific membrane granular markers on the cell surface or soluble granular markers in the extracellular medium. However, membrane markers do not appear on the cell surface if neutrophils secrete their products via “kiss and run” exocytosis [34], via budding of membrane vesicles [12–16], or via compound exocytosis [17]. Detection of soluble markers can be complicated in cases where they are secreted in the form of vesicles.

We performed proteome analysis of extracellular medium sampled from neutrophils that were attached to fibronectin for 20 minutes under different conditions [20, 21]. The proteins were extracted with chloroform-methanol (2 : 1) solution. This procedure allowed us to extract proteins that are secreted by cells both as individual molecules and as part of vesicles or cytonemes. During 20 minutes, the secreted proteins interacted with the proteases and other hydrolytic enzymes as well as with reactive oxygen species produced by the same neutrophils. As a result, the number of intact proteins that can be identified has decreased. For mass spectrometric identification of secreted proteins, we stained electrophoretic gels with the relatively low-sensitivity Coomassie Brilliant Blue dye, which allowed us to identify only a limited number of basic proteins. Nevertheless, this method made it possible to develop characteristic protein profiles of neutrophil secretion for various experimental conditions. Each protein profile was established by the results of three independent experiments.

**Figure 4:**
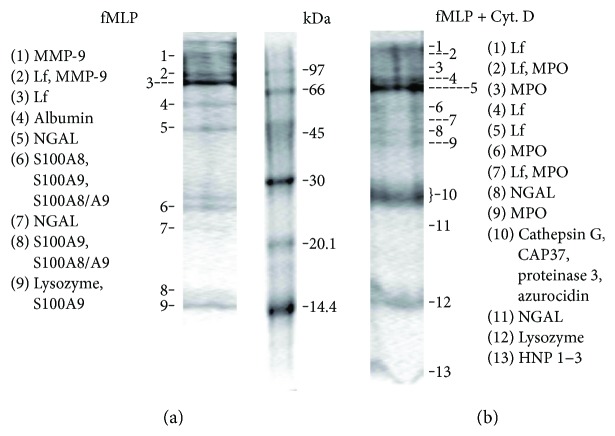
SDS-PAGE separation of proteins secreted by neutrophils upon adhesion to fibronectin in the presence of fMLP and cytochalasin D. Human neutrophils were attached to fibronectin-coated substrata for 20 min in the presence of 1 *μ*M fMLP taken separately (a) or in combination with 10 *μ*M cytochalasin D (b). Samples of extracellular medium were collected and proteins were extracted and, after concentration, were subjected to 15% SDS-PAGE. The gels were stained with Coomassie Brilliant Blue. The lines mark the places from which pieces of gel were cut. In these pieces, after trypsin hydrolysis directly in the gel, the designated proteins were detected by mass spectrometry. Pictures represent typical protein profiles, observed in three independent experiments for each EM example.

Upon adhesion to fibronectin, the control (resting) neutrophils secreted components of secretory vesicles (albumin), secondary (LF, NGAL, and lysozyme) and primary granules (only MPO), and cytosolic S100 proteins [20, 35] (Table 1, marked with a cross). The protein profile of fMLP-treated neutrophil secretion compared to that of control cells was enriched with the tertiary granule component MMP-9, or gelatinase B, 92 kDa (Figure 4(a), Table 1), an enzyme with an important role in neutrophil migration due to its capacity to degrade the extracellular matrix.

In the presence of cytochalasin D, the secretion of fMLP-treated neutrophils became enriched with the primary granule bactericides (such as MPO, cathepsin G, proteinase 3, CAP37, azurocidin, and HNP 1–3 or defensins 1–3), but the tertiary granule component MMP-9 did not fall in the number of major proteins (Figure 4(b), Table 1). In this case, the proteins were in the extracellular environment together with proteolytic enzymes (cathepsin G, proteinase 3), which were secreted by the same neutrophils (Table 1). During the incubation (20 minutes), proteins undergo partial proteolysis. This can explain the appearance of LF and MPO in the gel bands corresponding to different molecular masses (Figure 4(b)). The presence of NGAL in the two bands can be explained by the ability of this protein to exist as a monomer (Figure 4(a) band 7; Figure 4(b) band 11) or homodimer complex (Figure 4(a) band 5; Figure 4(b) band 8) [36, 37].

Like fMLP, PMA stimulated MMP-9 secretion in neutrophils (Table 2). The protein profile of secretion of neutrophils treated with PMA in combination with cytochalasin D included cathepsin G, the primary granule bactericide component, but not MMP-9 (Table 2).

The protein profile of neutrophils attached to fibronectin in the presence of LPS contained the same proteins as the control cells and additionally the following: (i) MMP-9, a tertiary granule component; (ii) cathepsin G and defensins, powerful bactericides of primary granules; and (iii) a number of cytosolic proteins such as actin, glycolytic enzyme glucose-6-phosphate dehydrogenase, and S100 proteins (Table 3). Lysozyme and actin were identified with low scores. Nevertheless, we include these proteins in the table, as they were identified in all our experiments. These data indicated that the adhesion of neutrophils to fibronectin in the presence of LPS was accompanied by the degranulation of the primary, secondary, and tertiary granules, as well as secretory vesicles. The in vivo release of LPS is considered an important mechanism for the induction of septic shock [36]. Products of LPS-induced degranulation of neutrophils contained powerful bactericides of primary and secondary granules that might strongly contribute to the damage of the surrounding tissues.

The protein profile of secretion of neutrophils that were attached to fibronectin in the presence of LPS in combination with cytochalasin D included lower number of identified proteins (Table 3). On the one hand, aggressive products of LPS-stimulated degranulation can modify secreted proteins thus complicating their identification. On the other hand, cytochalasin D substantially transformed the morphology of stimulated cells. The formation of intracellular vacuoles can occur as a result of compound or cumulative exocytosis [17] when secretory granules fuse with each other inside the cell. In this case, the release of granular content to the outside could be limited. Nevertheless, the protein profile comprising the major proteins of the extracellular medium of neutrophils treated with LPS in combination with cytochalasin D included cathepsin G, the bactericide of primary granules, but not MMP-9 (Table 3).

### 3.3. Effect of Cytochalasin D on Actin Cytoskeleton in fMLP-, LPS-, or PMA-Treated Neutrophils upon Adhesion to Fibronectin

In conclusion, we compared the actin cytoskeleton in fMLP-, PMA-, or LPS-stimulated neutrophils with the distribution of actin in cells stimulated with secretory stimuli in combination with cytochalasin D. For this purpose, the cells were attached to fibronectin during 20 min in the presence of tested drugs and fixed with paraformaldehyde and permeabilized with Triton X-100. Unfortunately, thin membrane structures, such as cytonemes or invaginations, were destroyed as a result of such treatment, and we could observe the distribution of actin only within the cells. The cells were then stained with fluorescent phalloidin, which binds to actin filaments much more tightly than the actin monomer so that actin filaments differ as particularly bright structures [9].

**Figure 5:**
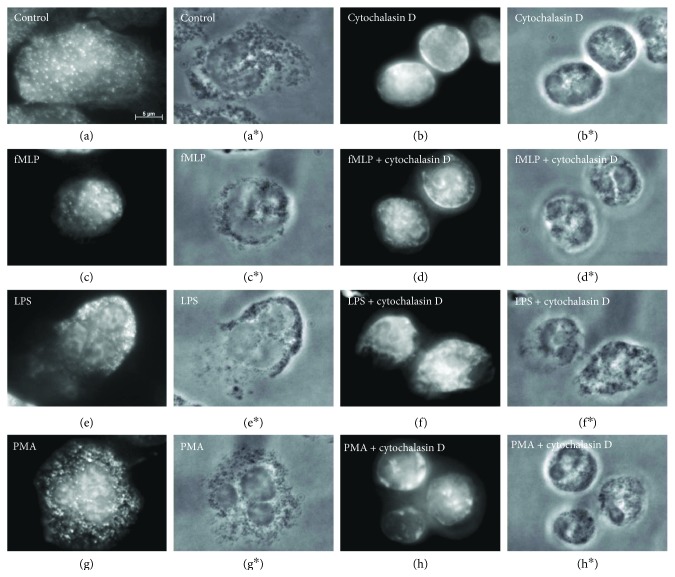
Fluorescent staining of actin cytoskeleton in neutrophils that were attached to fibronectin under various conditions. Fluorescent (a–h) and phase-contrast (a^∗^–h^∗^, resp.) images of cells that were attached to fibronectin-coated substrata under the control conditions (a) or in the presence of 1 *μ*M fMLP (c), 20 *μ*M LPS (e), or 0,1 *μ*M PMA (g), taken separately or in combination with 10 *μ*M cytochalasin D (b, d, f, h, resp.) for 20 min at 37°C. Neutrophils were stained for actin with FITC phalloidin. Pictures represent typical images observed in two independent experiments.

In control and fMLP-, PMA-, or LPS-stimulated cells, we observed small actin filaments that were distributed throughout the cell and were located closer to the base of the cells. The cytoplasm of cells was also diffusely stained (Figures 5(a), 5(c), 5(e), and 5(g)). In the presence of cytochalasin D, the staining for actin was diffuse with some redistribution of actin into the cortical layer. No noticeable filamentous structures in the cytoplasm of control and fMLP-, PMA-, or LPS-stimulated cells were observed (Figures 5(b), 5(d), 5(f), and 5(h)). These data show that in the presence of cytochalasin D, the actin cytoskeleton of the attached neutrophils was present in the depolymerized state, regardless of whether the cells were stimulated or not.

## 4. Discussion

Neutrophils undergo two types of secretion: a constitutive type of secretion typical for all types of cells and regulated secretion (degranulation) that occurs in response to stimulation [34]. For neutrophils in suspension granules are released in a hierarchical fashion in response to secretory stimuli as well as during in vivo exudation by first releasing secretory vesicles, followed by tertiary, secondary, and finally primary granules [1, 37, 38]. The adhesion of resting neutrophils to fibronectin was accompanied by the release of components of specific and primary (only MPO) granules and secretory vesicles but not by the secretion of MMP-9, a tertiary granule component [20]. MMP-9 secretion only occurred when neutrophil adhered to fibronectin in the presence of secretory stimuli such as fMLP, LPS, or PMA (Tables 1 [Table tab2]–3).

However, when the secretory stimuli were used in combination with cytochalasin D, MMP-9 ceased to be one of the major proteins of secretion (Tables 1 [Table tab2]–3). The similar inhibitory effect of cytochalasin D on the secretion of MMP-9 was previously observed in the PMA-stimulated human malignant glioma cells [39], osteoclasts [40], and monocytes [41]. Staurosporine, another actin-depolymerizing microbial alkaloid, also inhibited MMP-9 secretion in the TNF-stimulated neutrophils or in epidermal keratinocytes during hypoxia [42, 43]. The data taken together indicate that actin polymerization is critical for the secretion of MMP-9. Actin polymerization is required for multiple membrane fusion/fission events during exocytosis [6]. The inhibition of actin polymerization with cytochalasin D may interfere with docking or fusion of tertiary granules with the plasma membrane thereby inhibiting the release of MMP-9.

In addition to the suppression of the secretion of MMP-9, cytochalasin D evoked release of cathepsin G and other bactericidal agents contained in the primary granules in fMLP- or PMA-stimulated neutrophils (Tables 1 and 2). These data are in agreement with earlier reported works that revealed an obligatory role for F-actin disassembly for primary granule secretion in suspended cells [10, 11].

The presence of fMLP, LPS, or PMA upon adhesion modified the morphology of the attached neutrophils differently (Figures 1 [Fig fig2]–3). But in the presence of cytochalasin D, all stimulated and resting neutrophils exhibited a uniform non-spread shape and developed thread-like membrane tubulovesicular extensions (cytonemes) (Figures 1 [Fig fig2]–3). It indicated that the formation of cytonemes did not depend on cell stimulation, but on the actin cytoskeleton, which is a characteristic of the constitutive process. In the presence of stimuli (Figure 1(f), Figure 2(b), and Figure 3(b)), we observed partially destroyed, swelled, and shed from the cell cytonemes. Aware that cytonemes contain cathepsin G and other bactericide agents of primary and secondary granules [20, 21], we suggest that cytochalasin D-induced secretion of bactericides in fMLP- or PMA-stimulated neutrophils (Tables 1 and 2) occurs through the formation, shedding, and lysis of cytonemes, protrusions from a constitutive secretory trafficking. It is known that a certain level of basic secretion in various secretory cells is observed in the absence of stimulation. Apparently, some secretory proteins during of their biogenesis pass the secretory granules and follow the constitutive secretory pathway [34].

LPS taken separately initiated the secretion of primary granule components such as cathepsin G and defensins (Table 3) that was not accompanied by the formation of cytonemes (Figure 2(a)). In this case, we observed a regulated (in response to stimulation) secretion of bactericidal agents. The combination of LPS and cytochalasin D evoked the formation of cytonemes (Table 3, Figure 2(b)). In this case, both regulated and constitutive secretory pathways may contribute to the bactericide release.

Formation cytonemes under the influence of actin-depolymerizing alkaloids may occur due to blockage of the separation of secretory vesicles from the plasma membrane and from each other [20, 21, 27]. As a result, secretory trafficking extends outward the cells as cytonemes, which consist of membrane vesicles and tubes of the same diameter lined up in a row. GTPase dynamin in close cooperation with the actin cytoskeleton controls the process of separation of membrane vesicles from the initial membrane in intracellular transport processes. Dynamin oligomerizes into a helical polymer at the neck of budding vesicle, and dynamin oligomer constricts in the presence of GTP and catalyzes membrane fission leading to vesicle separation upon GTP hydrolysis. Actin filaments are thought to generate the force required for vesicle separation [44, 45]. Hypothesis about the mechanism of cytoneme formation was supported by the results of our work demonstrating that dуnasore, a specific inhibitor of dynamin GTPase activity, and cytochalasin D initiate the formation of the similar size and composition cytonemes in human neutrophils [20, 21].

Similar, cytochalasin D-induced invaginations in the neutrophil plasma membrane may appear due to the blocking of endocytic vesicle scission from the plasma membrane as a result of actin depolymerization. TEM studies of neutrophils treated with a combination of LPS and cytochalasin D showed structures resembling “inward» cytonemes—vacuoles interconnected into a chain ending with an opening in the plasma membrane” (Figures 2(d1)–2(d4)). In Hep-2 cells, cytochalasin D induced the formation of inward tubules measuring 120–220 nm in diameter and at least to 6 *μ*m in length [46], a similar size to the cytonemes of neutrophils.

In conclusion, cytochalasin D might impair extravasation and migration to the site of infection of neutrophils stimulated by the chemoattractant fMLP or bacterial LPS via (i) the inhibition of the secretion of MMP-9, a key player in the degradation of the vascular basement membranes and interstitial structures, and (ii) the simultaneous stimulation of the secretion of aggressive bactericidal agents which trigger the inflammation of the surrounding tissues (Tables 1 [Table tab2]–3). Actin depolymerization resulted in a change of neutrophil adhesion to the substrate and led to the formation of cytonemes which are able to make contact with the substrate or cells at a distance (Figures 1 [Fig fig2]–3). The binding of bacteria or fungi by cytonemes enriched with antibacterial agents may be an alternative to phagocytosis in the fight of neutrophil against bacterial and fungal pathogens [19, 22–24]. The secretion of alkaloids (toxins) capable of actin depolymerization can become an important factor in the invasion strategy of bacteria or fungi. Pathogens could manipulate the migration of neutrophils to the site of infection and their ability to bind and kill pathogens using such alkaloids.

## Figures and Tables

**Table 1:** A list of proteins that were secreted by neutrophils into the extracellular medium during a 20 min incubation with fibronectin-coated substrata under control conditions, in the presence of 1 *μ*M fMLP alone or in the presence of both 1 *μ*M fMLP and 10 *μ*M cytochalasin D (fMLP + Cyt. D). Mass spectrometric analysis data were taken from experiments with fMLP. Analogous proteins that were identified in control experiments or in experiments with fMLP + Cyt. D are marked (**+**). Mass spectrometric data for proteins that were identified upon fMLP + Cyt. D treatment were taken from these experiments. Proteins were separated by SDS-PAGE and identified by mass spectrometric analysis. ^∗^Protein was identified by MSMS analysis. Proteins identified in two or three analogous experiments were included in the list.

Entrez ID	Protein name			Peptides matched/total	Coverage, %	MOWSE score
	Control	fMLP	fMLP + Cyt. D			
*Granular proteins*						
GI:33285860		MMP-9		27/105	31	123
GI:168988718	**+**	Albumin		22/56	34	163
GI:186833	**+**	Lactoferrin	**+**	29/59	47	160
GI:7245433	**+**	NGAL	**+**	8/12	51	130
GI:13399627	**+**	Lysozyme	**+**	9/28	51	84
GI:7766942	**+**		MPO	24/89	52	114
GI:3891975			Cathepsin G	10/25	41	105
GI:227250			CAP37^∗^	—	18	77
GI:119589996			Proteinase 3^∗^	—	39	127
GI:2877			Azurocidin^∗^	—	12	95
GI:254839344			HNP 1^∗^	—	63	111
GI:75765819			HNP 2^∗^	—	62	95
GI:229858			HNP 3^∗^	—	63	111
*S100 proteins*						
GI:82407447	**+**	S100A8/A9		9/21	91	143
GI:4506773	**+**	S100A9		9/21	90	136
GI:21614544	**+**	S100A8		9/23	59	127

**Table 2 tab2:** A list of proteins that were secreted by neutrophils into the extracellular medium during a 20 min incubation with fibronectin-coated substrata under control conditions, in the presence of 0,1 *μ*M PMA alone or in the presence of both 0,1 *μ*M PMA and 10 *μ*M cytochalasin D (PMA + Cyt. D). Mass spectrometric analysis data were taken from experiments with PMA. Analogous proteins that were identified in experiments with PMA + Cyt. D are marked (+). Proteins were separated by SDS-PAGE and identified by mass spectrometric analysis. Proteins identified in two or three analogous experiments were included in the list.

Entrez ID	Protein name		Peptides matched/total	Coverage, %	MOWSE score
	PMA	PMA + Cyt. D			
*Granular proteins*					
GI:22532481	MMP-9		17/76	29	90
GI:332356380	Albumin		16/52	29	91
GI:1619857	Lactoferrin	+	34/67	50	225
GI:4261868	NGAL	+	14/56	68	109
GI:17942571	Lysozyme	+	5/14	53	73
GI:3891975		Cathepsin G	13/23	37	152
*S100 proteins*					
GI:82407447	S100A8/A9	+	15/116	90	79
GI:4506773	S100A9	+	6/29	57	78
GI:21614544	S100A8		5/14	53	73

**Table 3:** A list of proteins that were secreted by neutrophils into the extracellular medium during a 20 min incubation with fibronectin-coated substrata in the presence of 20 *μ*g/mL LPS alone, 10 *μ*M cytochalasin D (Cyt. D) alone, or in the presence of both 20 *μ*g/mL LPS and 10 *μ*M cytochalasin D (LPS + Cyt. D). Mass spectrometric analysis data were taken from experiments with LPS. Analogous proteins that were identified in experiments with LPS + Cyt. D are marked (+). Proteins were separated by SDS-PAGE and identified by mass spectrometric analysis. ^∗^Protein was identified by MSMS analysis. Proteins identified in two or three analogous experiments were included in the list.

Entrez ID			Peptides matched/total	Coverage, %	MOWSE score
	LPS	LPS + Cyt. D			
*Granular proteins*					
GI:269849668	MMP-9		22/69	30	225
GI:113576	Albumin		21/85	40	99
GI:85700158	Lactoferrin	+	23/71	41	241
GI:1171700	NGAL	+	10/28	60	134
GI:48428995	Lysozyme C		6/37	36	49
GI:129825	MPO	+	16/79	21	98
GI:115725	Cathepsin G	+	10/64	38	131
GI:30316322	Defensin HNP 1		6/8	20	75
GI:30316323	Defensin HNP 3		6/12	20	68
*Cytoskeleton proteins*					
GI:46397333	Beta actin	+	7/50	25	43
GI:54036678	Gamma actin	+	7/50	25	43
*Energy metabolism enzymes*					
GI:7669492	GAPDH^∗^		—	15	71
*S100 proteins*					
GI:115444	S100A9		8/37	81	94
GI:115442	S100A8		8/17	61	128

## Abbreviations

SEM: Scanning electron microscopy
TEM: Transmission electron microscopy
MPO: Myeloperoxidase
Lf: Lactoferrin
NGAL: Neutrophil gelatinase-associated lipocalin
GAPDH: Glyceraldehyde-3-phosphate dehydrogenase
S100A8, S100A9: The S100 calcium-binding proteins, known also as calgranulins A and B or myeloid-related proteins MRP8 and MRP14, or cystic fibrosis antigen
S100A8/A9: Calprotectin
MMP-9: Matrix metalloproteinase 9 or gelatinase B, 92 kDa
HNP 1–3: Human neutrophil peptides 1–3 or defensins
CAP37: Cationic antimicrobial protein 37
LPS: Lipopolysaccharide from *Salmonella enterica* serovar Typhimurium
fMLP: *N*-Formylmethionyl-leucyl-phenylalanine
PMA: Phorbol 12-miristate 13-acetate
CDT: *Clostridium difficile* binary toxin.
