# Erratum to “Mold Alkaloid Cytochalasin D Modifies the Morphology and Secretion of fMLP-, LPS-, or PMA-Stimulated Neutrophils upon Adhesion to Fibronectin”

**DOI:** 10.1155/2018/7202698

**Published:** 2018-07-05

**Authors:** Svetlana I. Galkina, Natalia V. Fedorova, Marina V. Serebryakova, Evgenii A. Arifulin, Vladimir I. Stadnichuk, Ludmila A. Baratova, Galina F. Sud'ina

**Affiliations:** ^1^A. N. Belozersky Institute of Physico-Chemical Biology, Lomonosov Moscow State University, Leninskie Gory, Moscow 119234, Russia; ^2^Physical Department, Lomonosov Moscow State University, Leninskie Gory, Moscow 119234, Russia

In the article titled “Mold Alkaloid Cytochalasin D Modifies the Morphology and Secretion of fMLP-, LPS-, or PMA-Stimulated Neutrophils upon Adhesion to Fibronectin” [1], there was an error in Figure 4, where lysosome should be corrected to lysozyme. The figure has been corrected in place.
